# Enhancing predictive analytics in mandibular third molar extraction using artificial intelligence: A CBCT-Based study

**DOI:** 10.1016/j.sdentj.2024.11.007

**Published:** 2024-11-26

**Authors:** Faezeh Khorshidi, Rasool Esmaeilyfard, Maryam Paknahad

**Affiliations:** a Computer Engineering and Information Technology Department, Shiraz University of Technology, Shiraz, Iran; b Oral and Dental Disease Research Center, Oral and Maxillofacial Radiology Department, Dental School, Shiraz University of Medical Sciences, Shiraz, Iran

**Keywords:** Artificial Intelligence, Natural Language Processing, Cone Beam Computed Tomography, Dental Radiology, Mandibular Third Molar Extraction

## Abstract

**Objective:**

Forecasting the complexity of extracting mandibular third molars is crucial for selecting appropriate surgical methods and minimizing postoperative complications. This study aims to develop an AI-driven predictive model using CBCT reports, focusing specifically on predicting the difficulty of mandibular third molar extraction.

**Methods:**

We conducted a retrospective study involving 738 CBCT reports of mandibular third molars. The data was divided into a training set consisting of 556 reports and a validation set containing 182 reports. The study involved two main steps: pre-processing and processing of the textual data. During pre-processing, the reports were cleaned and standardized. In the processing phase, a rule-based NLP algorithm was employed to identify relevant features such as angulation, number of roots, root curvature, and root-nerve canal relationship. These features were utilized for the training of a deep learning neural network to classify the extraction difficulty into four categories: easy, slightly difficult, moderately difficult, and very difficult.

**Results:**

The classification model achieved an accuracy of 95% in both the training and validation sets. Precision, recall, and F1-score metrics were calculated, yielding promising results with precision and recall values of 0.97 and 0.95 for the training set, and 0.97 and 0.89 for the validation set, respectively.

**Conclusion:**

The study demonstrated the high reliability of AI-based models to forecast the complexity of the mandibular third molar extractions from CBCT reports. The results indicate that AI-driven models can accurately predict extraction difficulty, thereby aiding clinicians in making informed decisions and potentially improving patient outcomes.

## 1 Introduction

The realm of dental radiology is experiencing a major shift due to the emergence of Artificial Intelligence (AI) technologies such as natural language processing (NLP) and deep learning. Numerous prior research projects have utilized artificial intelligence to detect different types of oral and maxillofacial conditions such as periodontal disease, dental caries, odontogenic cysts, and tumors. (Heo et al., 2021, Khanagar et al., 2022, Patil et al., 2022, Le et al., 2021, Esmaeilyfard et al., 2024, Esmaeilyfard et al., 2021, Paknahad et al., 2022). These developments have the ability to transform predictive analytics, allowing healthcare professionals to improve decision-making and enhance patient results. (Aljameel et al. 2023). One critical area poised to benefit from these innovations is the extraction of mandibular third molars, commonly known as wisdom teeth.

Accurate prediction of the difficulty involved in mandibular third molar extraction is essential for surgical planning and risk management. Utilizing predictive models, surgeons can estimate the complexity of the extraction before the procedure, allowing for better surgical planning (Sánchez-Torres et al. 2020). This foresight helps in selecting the most appropriate surgical approach, thereby reducing postoperative complications and improving overall treatment outcomes. Moreover, precise difficulty prediction aids in identifying high-risk patients, enabling surgeons to implement preventive measures and tailor their surgical strategies accordingly (Dong et al. 2024).

Utilizing NLP enables accurate retrieval of vital data from unstructured radiology reports, which deep learning models can then analyze (Young et al., 2019, Sorin et al., 2020, Donnelly et al., 2019, Huhdanpaa et al., 2018). This integration enhances the accuracy of diagnostics by minimizing human error and providing a more consistent analysis of radiological data (Büttner et al. 2023). Furthermore, AI-driven models can automatically analyze radiology reports and deliver rapid results to clinicians. This expedited diagnostic process reduces the time required for diagnosis and accelerates the initiation of appropriate treatment, ultimately benefiting patient care (Putra et al. 2022).

In this study, natural language processing (NLP) was instrumental in extracting critical features from radiology reports, including tooth angulation and root morphology. For a comprehensive discussion on the NLP methodology, challenges addressed, and the scoring system applied, please refer to Appendix A1. This study seeks to develop an AI-driven predictive model using CBCT reports, focusing specifically on the difficulty of mandibular third molar extraction.

## 2 Material and methods

All participants provided their informed consent. This study was conducted on a series of CBCT radiology reports of impacted mandibular third molar, encompassing a total of 738 cases. This sample size was determined based on the availability of complete data, the need to achieve a 95 % confidence level and 80 % statistical power, as well as the variability in features related to mandibular third molars (such as tooth angulation, number and curvature of roots, and their relationship to the mandibular canal). This sample size was selected to ensure the accuracy of the deep learning model and to avoid overfitting during the training process. The CBCT reports were sourced from oral and maxillofacial department of Shiraz dental university, providing a diverse patient dataset with consistent imaging protocols. The CBCT reports were collected over a period from January 2022 to July 2024. The inclusion criteria were reports that were specifically referred for mandibular third molar extraction and contained all the necessary information for the study including angulation, number of roots, curvature of roots, and relationship to the mandibular canal. Reports that lacked complete information were excluded. These reports were prepared by five different radiologists with minimum experience of the radiologists was 10 years. and were saved in Microsoft Word files. CBCT scans were conducted utilizing a NewTom VGi scanner (VGi EVO NewTom, Italy) with the specified settings: 8.9 s scan time, 5 mA, 19 mAs, 120 kV, and 10 × 10 cm field of view.

The collection of reports was used to identify the symptoms connected to the challenge of extracting the mandibular third molar in consultation with an oral and maxillofacial surgeon. This consultation helped determine the text processing procedure and the relevant clinical features. Through evaluating the reports, four primary features were identified: angulation, number of roots, curvature of roots, and relationship to the mandibular canal. The concepts related to these features were also determined. The four features—angulation, number of roots, root curvature, and relationship to the mandibular canal—were chosen based on previous research, clinical relevance, and expert consensus. These features are known to impact extraction complexity and were confirmed by specialists as essential for accurate prediction and practical application in surgical planning. Each feature was scored using a specific scale to quantify extraction difficulty:

*Angulation*: The angulation of the third molar relative to other teeth was scored on a scale from 1 to 4, reflecting difficulty levels based on orientation: Mesioangular (1), Horizontal (2), Vertical (3), Distoangular(4). Higher scores indicate more complex angulation types that can increase extraction difficulty.

*Number of Roots*: Root number was scored as: Single fused root (1), Two roots (2), Three or more roots (3). More roots generally indicate greater complexity in extraction.

*Curvature of Roots*: Root curvature was rated on a scale from 1 to 3, based on the complexity of each curvature type: Incomplete roots (1), Straight roots (2), Dilacerated roots (3). Higher scores denote increased difficulty due to challenging root shapes.

*Relationship to the Mandibular Canal*: Proximity to the mandibular canal was scored from 1 to 4: No contact (1), Approximation (2), Contact (3), Inside (4). Closer proximity to the canal, indicated by higher scores, raises the risk of complications and extraction difficulty. This structured scoring system quantifies each feature's impact on extraction difficulty, enabling the AI model to assess cases systematically. Each feature was linked to specific “concepts” encompassing synonyms and related phrases to capture variability in report language:

This approach ensured that linguistic variations in radiology reports were standardized, allowing for consistent data extraction across all reports.

The identified features were extracted from the textual data using a rule-based NLP algorithm. This process involved in NLP is discussed in Appendix A2.

Based on expert consultation, four classes of difficulty for mandibular third molar extraction were specified. The difficulty of mandibular third molar extraction is assessed using a scoring system, where different ranges correspond to specific difficulty classes. Scores between 4–6 are labeled as D1 (Easy), 7–8 as D2 (Slightly difficult), 9–10 as D3 (Moderately difficult), and 11–13 as D4 (Very difficult). These labels (D1 to D4) reflect the increasing complexity of extraction, with higher scores indicating more challenging procedures. The difficulty levels were categorized as easy, slightly difficult, moderately difficult, and very difficult based on the total scores of the identified features. Each report was classified into one of these categories using the predefined scoring system.

The rule-based NLP algorithm was validated by testing it on a subset of reports, which were cross-referenced with human-annotated data to ensure accuracy. Specifically, a sample of reports was manually annotated by dental radiology experts, highlighting key features like angulation, number of roots, curvature, and the relationship to the mandibular canal. The algorithm’s output was then compared to these expert annotations to assess its precision and reliability in feature extraction. This validation step confirmed that the NLP algorithm consistently identified relevant features, aligning closely with expert assessments.

### 2.1 Processing steps

**Fig. 1:**
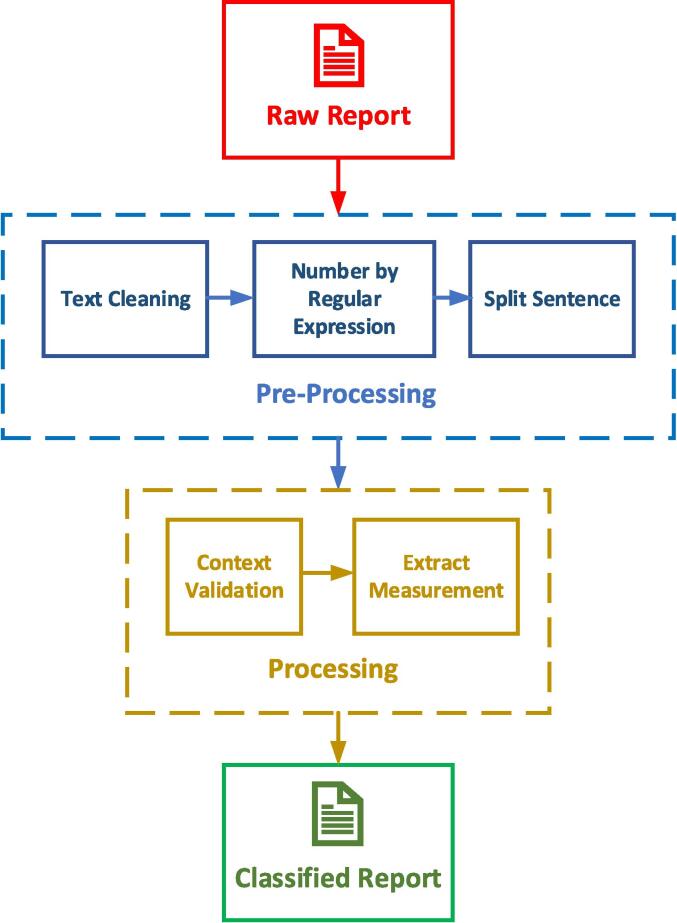
Overview of the processing steps.

An overview of the processing steps in this study is summarized in Fig. 1. The text processing of the reports was divided into two main steps: pre-processing and processing.

During the pre-processing phase, the raw reports underwent several data cleaning and transformation steps, utilizing the spaCy library for tasks like tokenization and sentence separation. For more detailed information on the pre-processing methodology, including the specific machine learning techniques and tools used, please refer to Appendix A3.

In the processing phase, a rule-based NLP algorithm was used to standardize and extract key features from the reports, addressing variability in terminology among radiologists. For a comprehensive explanation of the processing methodology, including synonym handling and scoring criteria, please refer to Appendix A4.

**Fig. 2:**
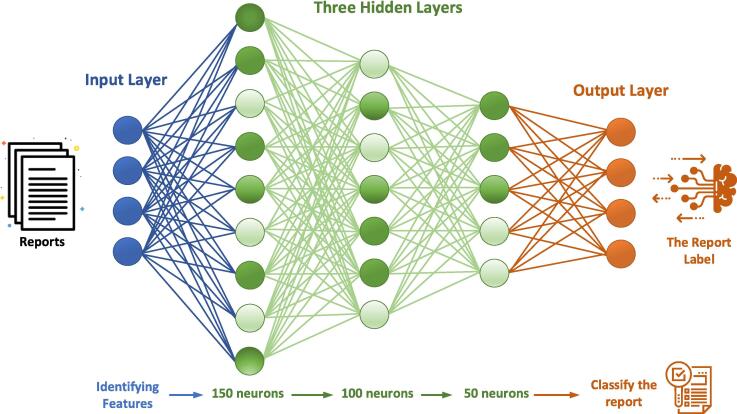
Structure of the Deep Learning Neural Network.

**Fig. 3:**
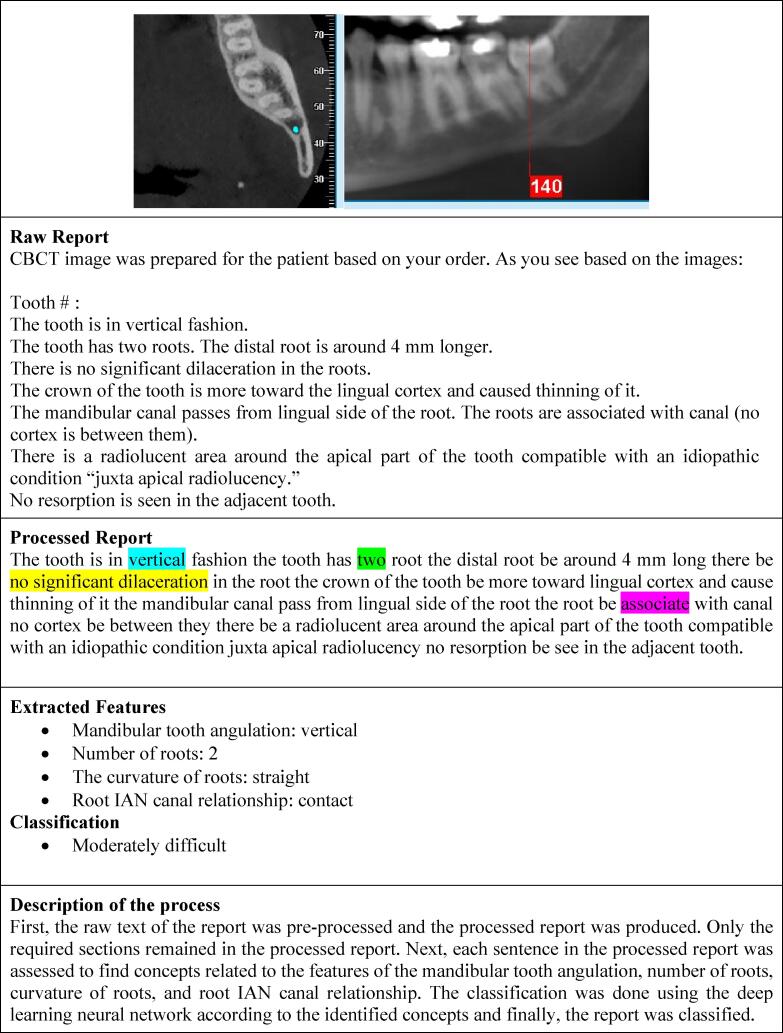
Example of Information Extraction and Report Classification Process in a Sample Patient.

The neural network architecture, as illustrated in Fig. 2 and detailed in Appendix A5, was designed with four input neurons corresponding to the key extracted features, along with multiple hidden layers to classify extraction difficulty. Fig. 3 presents an example of the information extraction and classification process, showcasing the model’s capability in dental radiology analytics. For a full explanation of the network’s structure, training parameters, and overfitting prevention methods, please refer to Appendix A5.

Three key metrics— recall, precision, and F1-score—were used to assess the accuracy of the text processing for each class (label). These metrics are discussed in Appendix A6.

## 3 Results

**Fig. 4:**
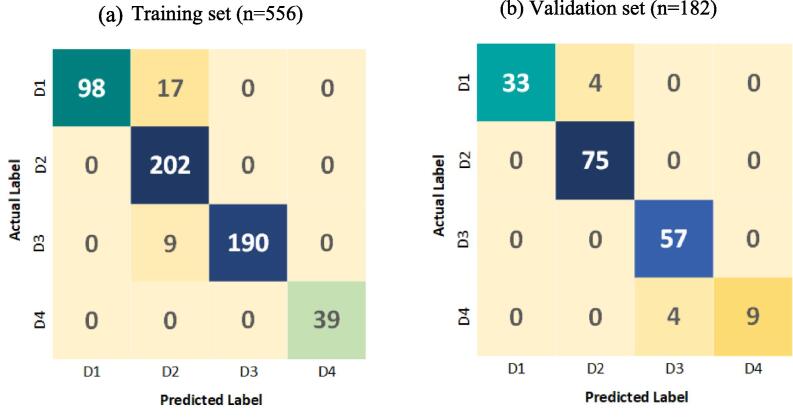
Confusion Matrices of the Classification.

This study split 738 cases into a training group comprising 556 examples and a validation group consisting of 182 examples., in order to assess the model's capacity to perform effectively on new data. The classification algorithm obtained a 95 % accuracy in both sets. To further evaluate how well the model performed, confusion matrices were created for every level of difficulty, as depicted in Fig. 4. The rows in the matrices represent the genuine difficulty levels, and the columns display the model's anticipated classes. This enables a direct comparison between accurate and inaccurate classifications, offering a comprehensive understanding of the model's capacity to classify cases correctly.

The results for each mandibular third molar extraction difficulty label in the training as well as validation datasets are presented in Table 1.

**Table 1:** Precision, Recall, and F-score.

Training Set	Precision	Recall	F-score
D1	1.00	0.86	0.92
D2	0.88	1.00	0.94
D3	1.00	0.95	0.97
D4	1.00	1.00	1.00
Total	0.97	0.95	0.96
Validation Set	Precision	Recall	F-score
D1	1.00	0.89	0.94
D2	0.95	1.00	0.97
D3	0.93	1.00	0.97
D4	1.00	0.67	0.80
Total	0.97	0.89	0.92

Mistakes in report handling were classified into three categories: concept errors, classification errors, and spaCy errors. Table A3 in the Appendices offers an overview of these mistakes for both the training and validation datasets. A total of 15 errors were discovered in the training set and 6 errors were identified in the validation set.

## 4 Discussion

AI models developed for analyzing dental radiology reports can serve as valuable educational tools. These models can be used to train dental students and novice surgeons, helping them improve their skills in interpreting radiological data and planning surgical procedures(Thurzo et al. 2023). Additionally, AI systems can provide data-driven clinical recommendations, assisting healthcare providers in making better-informed treatment decisions. This support system enhances clinical decision-making, ensuring that patients receive optimal care based on comprehensive data analysis (Carrillo‐Perez et al. 2022). The aim of the current study was to enhance predictive analytics in dental radiology reports using artificial intelligence, specifically focusing on the classification of mandibular third molar extraction difficulty through NLP. The methodology utilized in this study involved a classification algorithm that analyzed the semantic meaning and interaction between words and phrases within the maxillofacial radiology reports.

The AI-based model enhances clinical workflow by preemptively identifying difficult cases based on anatomical features before the radiologist’s report. This allows clinicians to perform better risk assessments and tailor surgical strategies accordingly, improving decision-making and reducing potential complications. Rather than duplicating the information in the CBCT report, the AI tool provides valuable support in streamlining pre-surgical planning, thus increasing both accuracy and efficiency in clinical practice.

For further details on the advantages of our rule-based NLP method and how it enhances the precision of feature extraction compared to conventional text analysis techniques, please refer to the Appendix A7.

In this study, we achieved a 95 % accuracy rate for both the training and validation sets, demonstrating the high reliability of NLP in classifying free-text maxillofacial radiology reports. The methods employed to extract key features—angulation, number of roots, curvature of roots, and root-IAN canal relationship—yielded promising results, with high precision, recall, and F1-score metrics. This level of accuracy is comparable to other studies conducted on pathology and radiology reports. For example, Alex et al. (2019) reported high accuracy in diagnosing brain image reports, achieving 96 % for labels, 95 % for entities, 98 % for relations, and 94 % for negation. Gálvez et al. (2017) also achieved a 97 % accuracy rate, correctly identifying 140 out of 144 pediatric diagnostic radiology reports for deep venous thrombosis in children.

Similarly, Brown and Kachura (2019) reported an accuracy of 92 % in detecting acute ischemic stroke in brain MRI reports, outperforming other methods. Kim et al. (2019) demonstrated an impressive 98 % accuracy by utilizing a decision tree to detect acute ischemic stroke in brain MRI reports. These comparisons underline the effectiveness of our approach and its alignment using cutting-edge techniques in the field. The high accuracy metrics across different medical domains highlight the potential of our rule-based NLP method to provide reliable and actionable insights in maxillofacial radiology, thereby enhancing clinical decision-making and improving patient outcomes.

For a detailed overview of the study's strengths, challenges in data processing, and the strategies employed to address variability in linguistic features and reporting styles, please refer to the Appendix A8.

## 5 Conclusion

This study demonstrated the effectiveness of using natural NLP and AI to enhance predictive analytics in dental radiology, specifically for classifying the difficulty of mandibular third molar extractions. By employing a multi-step, rule-based text processing method, the study achieved high accuracy, precision, recall, and F1-scores, highlighting the reliability of this approach. The integration of NLP and AI in dental radiology shows substantial opportunity to enhance clinical judgment and patient results. The methods developed in this study provide a solid foundation for further advancements, paving the way for more accurate and automated analysis of radiology reports. This research marks a step forward in leveraging AI capabilities to enhance dental practice and improve patient care.

## Ethical approval

The ethics committee approved this study (IR.SUMS.DENTAL.REC.1401.092).

## Author Contributions

**Faezeh Khorshidi**: Conducted the experiments, developed the software and algorithms, and contributed to the writing of the original draft.

**Rasool Esmaeilyfard**: Contributed to the writing of the original draft, prepared the figures, managed the project, and provided guidance and critical revisions of the manuscript.

**Maryam Paknahad**: Led project administration, provided supervision and consultation, and performed the final revisions and editing of the manuscript.

## Declaration of Competing Interest

The authors declare that they have no known competing financial interests or personal relationships that could have appeared to influence the work reported in this paper.

## Appendices

The following are the Appendices to this article:
