# Supplementary material for: Enhancing predictive analytics in mandibular third molar extraction using artificial intelligence: A CBCT-Based study
Source: Saudi Dent J. 2024 Nov 26;36(12):1582–7. doi: 10.1016/j.sdentj.2024.11.007 (PMC11976095; doi:10.1016/j.sdentj.2024.11.007)
Supplement: Supplementary Data 1 [file mmc1.docx]

Appendices

# Appendix A1: NLP Methodology, Challenges, and Scoring Criteria for Feature Extraction

NLP plays a crucial role in extracting and interpreting valuable information from free-text radiology reports, transforming unstructured data into analyzable content. In this study, NLP is used to identify key features related to mandibular third molar extraction difficulty, such as tooth angulation and root proximity to the mandibular canal. By standardizing and analyzing linguistic variability in radiology reports, NLP enables consistent data extraction, which is essential for accurate AI-driven predictive modeling and supports more reliable clinical decision-making. AI applications in dental radiology face unique challenges, including the variability in report language and inconsistencies in radiological image interpretations. Dental radiology reports often contain diverse terminology and subjective descriptions, which complicates the extraction of standardized features. Additionally, interpreting CBCT images for mandibular third molar extractions involve detailed anatomical nuances that require precise analysis. Our approach addresses these challenges by employing a rule-based NLP model tailored to standardize terminology, ensuring consistent feature extraction. This innovation enhances the reliability of AI predictions, enabling more accurate modeling and analysis.

This AI-driven model offers distinct benefits to various stakeholders in dental healthcare. For oral surgeons and general dentists, it provides an objective tool to assess extraction difficulty, allowing for better surgical planning and reduced risk of complications. Educators can use this model as a training tool, offering dental students and trainees a valuable resource for understanding radiographic analysis and decision-making in complex cases. Additionally, for patients, this model supports improved surgical outcomes and a more informed consent process, as practitioners can provide clearer expectations regarding the complexity of the procedure. The AI model provides targeted benefits to oral surgeons, general dentists, and dental educators. For oral surgeons, it enables accurate preoperative planning, helping to minimize complications and enhance patient outcomes. General dentists benefit from decision-support for case selection and referrals, optimizing patient care. Dental educators can use the model for case-based learning, training students in CBCT interpretation and complex case management, thus building their confidence and skills in clinical decision-making.

Unlike conventional imaging modalities such as panoramic X-rays, CBCT provides high-resolution, three-dimensional images, enabling detailed visualization of anatomical structures.This accuracy is crucial for identifying complex features like tooth angulation, root morphology, and the proximity of roots to the mandibular canal, which significantly impact extraction difficulty. Thus, CBCT allows for more precise analysis, supporting better-informed clinical decisions. Current clinical approaches often rely on visual assessments and subjective interpretations, which can vary between practitioners and may lack precision. Predictive models based on AI have the potential to bridge these gaps by providing consistent, data-driven assessments. These models can identify high-risk cases more reliably, aiding clinicians in surgical planning and potentially reducing postoperative complications such as nerve damage or delayed healing. Accurate prediction models can help mitigate several postoperative complications associated with mandibular third molar extractions. These include nerve damage, which may result in numbness or altered sensation in the lower lip and chin, prolonged healing times, and increased risk of infection. By precisely estimating the complexity of each case, AI-driven models allow clinicians to tailor their surgical approach, potentially reducing these complications and enhancing overall patient recovery and satisfaction.

# Appendix A2. NLP Steps

***Tokenization:*** The spaCy library in Python was used for tokenization, breaking down the text into individual words or tokens. SpaCy’s tokenizer was chosen for its accuracy in handling medical terminology and complex text structures, enabling precise segmentation of radiology report content.

***Normalization:*** A normalization process was applied to standardize terminology across reports. This included converting synonyms and varied phrases into a consistent format. For example, terms like “tilted” or “angled” were mapped to standardized angulation descriptors. This step ensured that linguistic variations did not hinder feature extraction.

***Feature Extraction****:* Key features such as angulation, number of roots, root curvature, and relationship to the mandibular canal were identified using regular expressions and predefined patterns. Each feature had associated synonyms and descriptive phrases that were recognized through custom rules, enabling the algorithm to extract relevant information across diverse report styles.

A multilayer neural network (MLNN) was selected for this task due to its ability to model complex, nonlinear relationships among input features. Given the intricate anatomical data captured in CBCT reports, an MLNN was well-suited for learning patterns and dependencies that influence extraction difficulty. The network’s layered structure also allows for effective handling of diverse data representations, supporting accurate classification across varying levels of complexity.

# Appendix A3. Pre-processing

In the pre-processing step, the raw reports were transformed using machine learning techniques. The contents were extracted from Microsoft Word files, and headers and footers were removed. Text ambiguities were resolved with a sectionizer, followed by data cleaning steps like removing unwanted characters, tokenization, root word extraction, and sentence separation using the spaCy library. Numbers were also extracted, and sentences were split, preparing the text for further processing. The NLP algorithm was developed using Python, specifically leveraging the spaCy library, which is widely used for natural language processing tasks due to its efficiency and flexibility. SpaCy was chosen for its advanced tokenization, part-of-speech tagging, and dependency parsing capabilities, which are essential for accurately extracting and standardizing features from radiology reports. Additionally, regular expressions were used for identifying specific terms and synonyms associated with each feature. This setup offers a robust framework for replicating the NLP-based feature extraction process in similar studies.

# Appendix A4. Processing Step

In the processing step, an NLP algorithm based on rules was employed to derive relevant concepts from the texts to identify the four key features (Table 1). Since different radiologists used various synonyms for the same concepts, a set of synonyms was identified and standardized for each concept to unify the extraction process. For instance, in establishing the connection between the root and the nerve canal, the concept of “contact” was adapted to include synonyms like “association” and “no cortex.” These sets of synonyms for each concept were transformed into regular expressions, as listed in Table A1 in Appendices.

Given the presence of numerical data, such as measured distances, it was crucial to accurately determine and extract the number of roots. The number of roots, ranging from one to four, needed to be placed near the word "root" and not contain units like cm or mm, ensuring it wasn't a measured distance. Regular expressions were used to identify and extract these concepts from the texts, as shown in Table A2 in Appendices.

A radiologist assessed the degree of difficulty of each report based on the scores of the features and their concepts and assigned a score to each report. These scores were then converted into a label for each report, forming a new database. Each record in this database included the four mentioned features and the label of each report.

# Appendix A5. Deep Learning Model

The neural network’s input layer consists of four neurons, each representing one of the key features scored during the feature extraction process: angulation, number of roots, curvature of roots, and relationship to the mandibular canal. Each neuron receives the quantified score for its respective feature, allowing the network to process these inputs collectively and make predictions about extraction difficulty based on the combination of these anatomical factors. A deep learning multilayer neural network was used to classify the reports and automatically assign labels. This neural network had four input neurons, which matched the four features taken from each report. It consisted of three concealed layers comprising of 150, 100, and 50 neurons, respectively. The output layer consisted of four neurons, representing the four levels of extraction difficulty (corresponding to each class label). The activation function utilized was the hyperbolic tangent function, and the network was trained over a maximum of 300 iterations. The hyperbolic tangent (tanh) function was selected as the activation function due to its ability to model both positive and negative outputs, which is advantageous in classification tasks involving complex, varied data. Unlike the sigmoid function, which outputs values between 0 and 1, the tanh function produces outputs ranging from -1 to 1, allowing for better differentiation of feature scores. This range helps the model capture subtle distinctions in extraction difficulty, making tanh especially suitable for tasks requiring nuanced classification.

The predicted labels generated by the neural network were compared to the expert-assigned labels to determine the accuracy of the model. This comprehensive approach ensured the robust classification of CBCT reports, enhancing predictive analytics in dental radiology.

To prevent overfitting, we applied dropout regularization to the neural network, randomly deactivating a portion of neurons during training to improve generalization. A batch size of 32 was used to balance computational efficiency with stable gradient updates. The learning rate was set at 0.001, ensuring gradual adjustments during optimization. The Adam optimizer was chosen for its adaptive learning rate capabilities, allowing the model to converge more effectively compared to standard gradient descent methods. These settings collectively enhanced the model’s performance and robustness on new data.

# Appendix A6. Evaluation Metrics

The model’s accuracy was calculated as the proportion of correct predictions out of the total predictions made across all classes, providing a basic measure of overall performance. In addition to accuracy, we also assessed the model using precision, recall, and F1 score metrics to gain a more comprehensive evaluation of its classification ability.

Precision measured the proportion of true positive predictions out of all positive predictions made by the model, indicating its accuracy in identifying specific extraction difficulty levels. Recall represented the proportion of true positive cases correctly identified out of all actual cases, reflecting the model's ability to capture each difficulty level without missing cases. F1 Score, the harmonic mean of precision and recall, provided a balanced metric to account for any trade-offs between precision and recall.The precision, recall, and F1-score metrics were calculated using the following equations.

| $Accuracy =\frac{TP + TN}{P + N}$ | (1) |
| --- | --- |
| $Precision = \frac{TP}{TP + FP}$ | (2) |
| $F_{1} = 2\times\frac{Precision \times Recall}{Precision + Recall}$ | (3) |

Where P, N, TP, TN, and FP represent the number of positive cases, negative cases, correctly classified positive cases, correctly classified negative cases, and wrongly classified positive cases, respectively.

These metrics were calculated for each difficulty level (class) and averaged to give a clearer understanding of the model's performance across varying levels of extraction complexity.

# Appendix A7. Advantages of the Rule-Based NLP Approach in Analyzing Dental Radiology Reports

This approach differs from conventional automatic text analysis methods, which typically evaluate the entire text in a single step. Instead, our method involved multiple stages of analysis before reaching the final classification. This multi-step process allowed for a more detailed and accurate extraction of relevant features, ensuring a higher level of precision in the final analysis. A significant advantage of our approach is its reliance on rule-based techniques, which are particularly effective in this context. The factors influencing the difficulty of mandibular third molar extraction are well-known to surgeons, and diagnoses are typically based on established rules. Therefore, using a rule-based NLP method aligns well with these diagnostic criteria, allowing for more accurate classification outcomes. The use of rule-based methods, as employed in this study, leverages these established diagnostic criteria effectively, differentiating our approach from generalized automatic text analysis methods. These conventional methods might create a combination of rules but do not provide the specificity and accuracy required for this particular classification task. By focusing on these known factors and implementing a structured, rule-based analysis, our method enhances the accuracy and reliability of classifying the difficulty of mandibular third molar extractions from maxillofacial radiology reports. This advancement demonstrates the potential of AI-driven predictive analytics in improving clinical decision-making and patient outcomes in dental radiology.

# Appendix A8. Challenges and Strengths of the Study

This study exhibited several notable strengths. Firstly, the extraction and consideration of synonyms for the selected concepts significantly enhanced the study's accuracy. Given the variability in the vocabulary used by different radiologists to describe features, accounting for synonyms allowed for a more precise and consistent analysis, addressing a critical challenge in NLP applications. This attention to linguistic variability is one of the greatest strengths of this study, as it substantially increased the classification accuracy.

Secondly, the study effectively addressed the limitations posed by the available dataset size. While automatic text analysis methods typically require large datasets to function effectively, the specialized rule-based processing method developed in this study proved effective even with a limited number of reports. This underscores the importance of domain-specific knowledge and the creation of tailored rule-based systems when data is scarce, demonstrating that high accuracy can still be achieved with expert-driven approaches.

Thirdly, free-text maxillofacial radiology reports contain numerous context-specific terms that are crucial for accurately describing conditions or diseases. Properly associating these concepts with their relevant contexts, including handling negations and numerical values, is a complex task that requires meticulous attention. This study’s careful and precise handling of context-related terms ensured that the extracted features were accurately represented, contributing to the overall robustness and reliability of the classification model. This meticulous approach to context management is critical for ensuring the relevance and accuracy of the analysis, highlighting another key strength of the study.

The results of the current study revealed various errors that led to the misclassification of the difficulty of mandibular third molar extraction. Differentiating between errors occurring in the preprocessing and processing steps proved challenging, as these steps are often interdependent. For instance, errors in sentence separation might stem from insufficient training on medical documents or variations in reporting styles by radiologists. Reports written in Microsoft Word often contained issues such as extra spaces or separators, which were partially addressed during preprocessing. However, these anomalies can still impact the accuracy of text processing. The complexity of medical texts adds another layer of difficulty. When different concepts are mentioned in a single sentence (e.g., 'the tooth is in a mesioangular position with two separate roots'), accurately parsing and understanding the text becomes challenging. Similarly, when concepts are expressed in separate sentences, it complicates the processing procedures.

To address these challenges and improve the accuracy of NLP in classifying dental radiology reports, the standardization of report formats is recommended. Implementing a shared vocabulary can reduce the variability in terminology, thereby minimizing the number of synonyms and enhancing the consistency of the reports. Encouraging the use of standardized templates for writing radiology reports can help ensure that related phrases and concepts are clearly and consistently presented. This would reduce ambiguities and improve the accuracy of text processing. Expanding the training data to include a wider variety of medical documents can help the NLP algorithms better understand and process the specific nuances of medical texts. Training the model on reports from various radiologists with different styles can improve its ability to handle variations in expression. Developing more sophisticated preprocessing techniques to handle common text anomalies (e.g., extra spaces, separators) can enhance the quality of the input data. Implementing methods for better contextual understanding, especially in sentences where multiple concepts are presented, can improve the accuracy of feature extraction. Establishing a feedback loop where the model's performance is continuously monitored and improved based on new data and errors identified can further enhance the reliability and accuracy of the NLP classification system.

This study had some limitations, the foremost being the limited number of reports. Additionally, the data were sourced from only one center, and the learning was based solely on this dataset. Consequently, the findings may not be fully generalizable. Future studies should aim to include larger samples from multiple centers to enhance the robustness and generalizability of the results. This broader approach would provide a more comprehensive understanding and validation of the methodologies employed, ensuring that the findings are applicable across different settings and populations.

Table A1. Synonyms for Concept Extraction

| Concept | Synonyms |
| --- | --- |
| Incomplete root | Developing \| open apex \| not completely formed \| developed |
| Dilaceration | Curve \| curvature |
| Straight | No dilacerations |
| No contact | No association \| far from \| safe distance |
| Approximation | Proximity \| close proximity |
| Contact | Association \| in contact \| cannot be followed \| the cortex of the canal \| no cortex |

Table A2. Regular Expressions for Concept Extraction

| Concept | Regular expression |
| --- | --- |
| Mesioangular | Mesio.*(angular)* |
| Horizontal | Horizontal |
| Vertical | Vertical |
| Distangular | Distoangular |
| Number of roots | ([1-4] \| one \| two \| three) (?=.*(root(s)*)) |
| Incomplete root | Incomplete \| open apex \| develop \| not completely formed |
| Dilacerated | Dilacerations \| curvature \| caurve |
| Straight | (no)+.*(dilaceration) |
| No contact | No association \| not associated \| far from \| any contact \|  not in contact \| safe distance |
| Approximation | Proximity \| close proximity |
| Contact | Associate \| association \| contact \| not be follow \| no cortex |
| Inside | Inside |

Table A3. Errors by Category for the Training and Validation Sets

| Error group | Error type | Description | Training  (n=556) | Validation  (n=182) |
| --- | --- | --- | --- | --- |
| Concept | Concept mismatch | Two different concepts for the same feature | 9 | 0 |
| SpaCy | Wrong number extraction | Error in determining the number of tooth roots | 0 | 6 |
| Classification | Error in label prediction | The label was not correctly predicted by the model | 12 | 6 |
